# Supplementary material for: Performance Efficiency of Conventional Treatment Plants and Constructed Wetlands towards Reduction of Antibiotic Resistance
Source: Antibiotics (Basel). 2022 Jan 16;11(1):114. doi: 10.3390/antibiotics11010114 (PMC8773441; doi:10.3390/antibiotics11010114)
Supplement: Supplementary file 1 [file antibiotics-11-00114-s001.zip › antibiotics-1507046-supplementary.pdf]

Supplementary material

Performance Efficiency of Conventional Treatment Plants and Constructed Wetlands towards reduction of Antibiotic Resistance

Moushumi Hazra[1*], Lisa Durso[2]
[*1]Institute Post-Doctoral Fellow, Department of Hydrology, Indian Institute of Technology, Roorkee, Uttarakhand, India, [#2] Research Microbiologist, United States Department of Agriculture, Lincoln, NE
Corresponding author: Moushumi Hazra [1*]
Email: moushmihazra@gmail.com /moushumi.pd@hy.iitr.ac.in

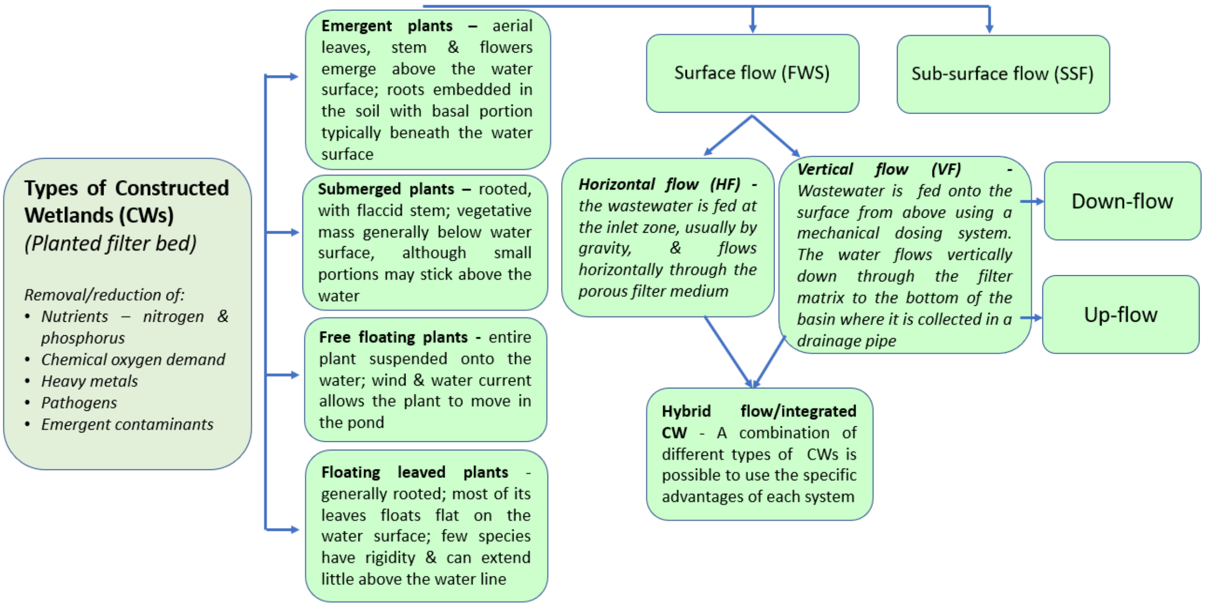

Figure S1: Types of Constructed wetlands according to their designs and specifications

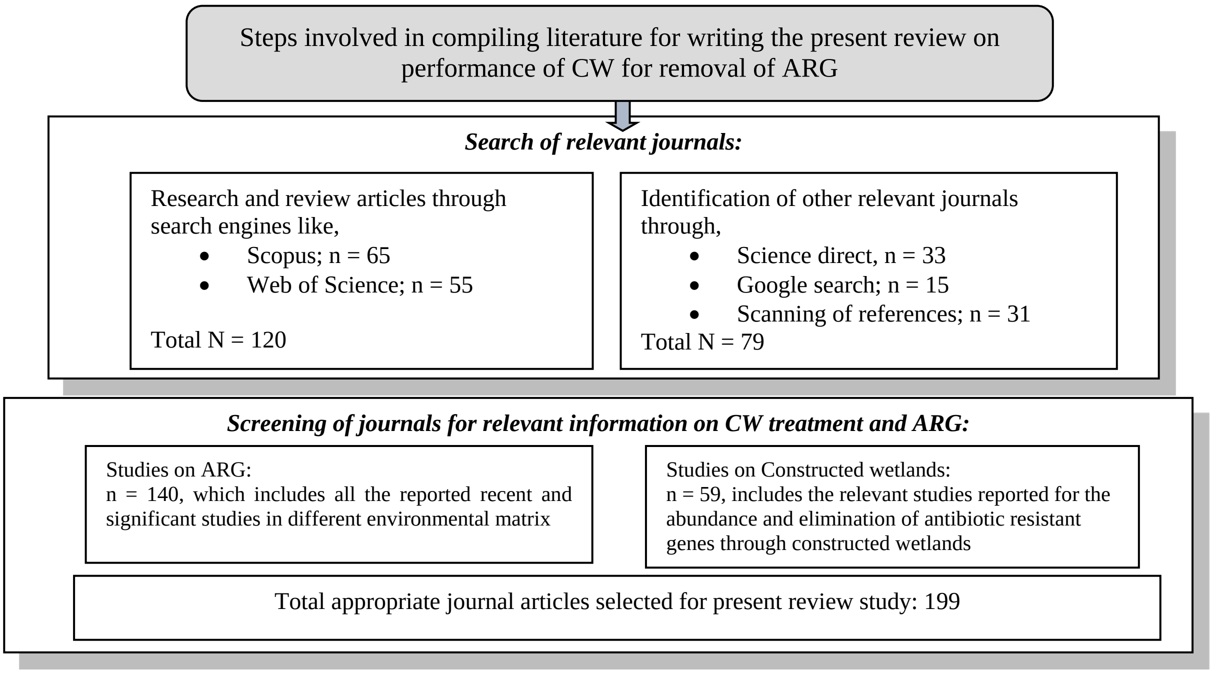

Figure S2: Flow chart to demonstrate the steps involved for writing the present review

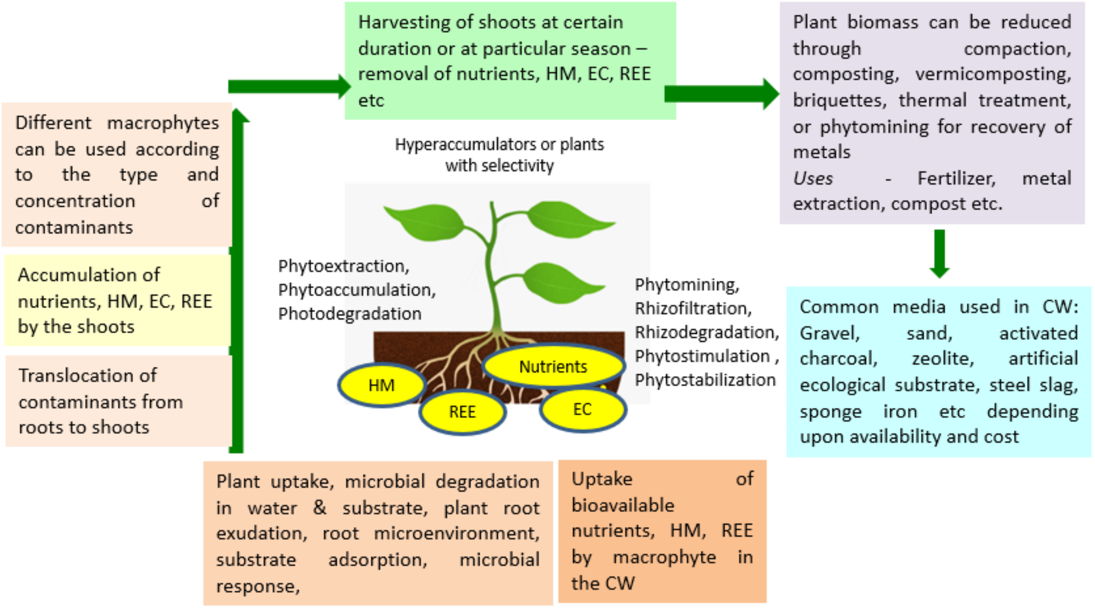

Figure S3: Role of macrophytes in Constructed wetlands (*Note*; HM-heavy metals; EC-emerging contaminants; REE-rare earth metals; CW-constructed wetlands)

Table S1: Comparative techno-economical assessment of target technologies based on suitability of reuse

Parameter	Target compounds	Technology		
		**CW**	**MBR**	**Activated Sludge**
Performance efficiency (%)	Pharmaceuticals	0–99%	<0–99%	94–98%
	Antibiotics	0–100%	<0–99%	90%
Need of post-treatment		Yes/No	No	Yes
Complexity in operation		Requires maintenance Ease in operation/management	Technical and skilled staff required. High process automation	Skilled staff required
Complexity in lay out/Ease of construction		Easy to construct, also commercially available	Commercially available	Construction needed, treats carbon and nitrogen compounds
Flexibility/reliability		Not flexibility, and not possible to change the design and geo-textile materials. Resilient to fluctuations in flow and shock loads. Affected by temperature	Flexibility in design with modular setup. Resilient to inflow fluctuations and shock loads	Not resilient to disturbances and toxic loading. Affected by pH, temperature, oxygen, nitrite, ammonia concentration
Footprint		Large areas for construction are required	Feasibility in reduction of space possible, Small footprint	Large area required
Environmental aspects (waste production, disposal, chemicals)		Pretreatment may be required based on the type of WW to avoid clogging. No post treatment required. Treated effluent can be directly discharged to water bodies. Sludge generated from primary treatment may be used as fertilizer with post treatment such as by adding alum	Treatment of concentrate and sludge required	Efficient for treating industrial wastewater
Investment cost		Very low as locally available plant species and substrate can be used. Demolition waste, bricks, marble remains can also be used	Membrane costly approximately 40 – 60% of total capital costs. Chemicals are required for membrane cleaning	Cost related to construction and electricity consumption
Other factors that assist in maximizing the performance efficiency for removal of ARB/ARG		Flow pattern governs the removal of ARB/ARG. Efficient in treating ARG and pathogens	Membrane needs to be replaced if fouling takes place to increase the performance efficiency. Accumulation of suspended colloidal particles on membrane. Better performance for microbial removal and for *int1* and ARGs	Sludge contains ARG that gets disseminated in soil and finally water bodies
Use in treatment technologies		Used as tertiary treatment or for polishing of effluents	Used as tertiary treatment	Used for secondary treatment
